# Magnetic properties of fluffy Fe@*α*-Fe_**2**_*O*_**3 **_core-shell nanowires

**DOI:** 10.1186/1556-276X-8-423

**Published:** 2013-10-17

**Authors:** Xiaobing Cao, Weihua Wang, Xinghua Zhang, Luyan Li, Yahui Cheng, Hui Liu, Sichao Du, Rongkun Zheng

**Affiliations:** 1 Department of Electronics & Key Laboratory of Photo-Electronic Thin Film Devices and Technology of Tianjin, Nankai University, Tianjin 300071, China; 2 School of Material Science and Engineering, Hebei University of Technology, Tianjin 300130, China; 3 School of Science, Shandong Jianzhu University, Jinan 250101, China; 4 School of Physics, The University of Sydney, Sydney, NSW 2006, Australia

**Keywords:** Fe@*α*-Fe_2_O_3_ core-shell nanowires, Coercivity, Exchange bias

## Abstract

Novel fluffy Fe@*α*-Fe_2_O_3_ core-shell nanowires have been synthesized using the chemical reaction of ferrous sulfate and sodium borohydride, as well as the post-annealing process in air. The coercivity of the as-synthesized nanowires is above 684 Oe in the temperature range of 5 to 300 K, which is significantly higher than that of the bulk Fe (approximately 0.9 Oe). Through the annealing process in air, the coercivity and the exchange field are evidently improved. Both the coercivity and the exchange field increase with increasing annealing time (*T*_*A*_) and reach their maximum values of 1,042 and 78 Oe, respectively, at *T*_*A*_ = 4 h. The magnetic measurements show that the effective anisotropy is increased with increasing the thickness of the*α*-Fe_2_O_3_ by annealing. The large values of coercivity and exchange field, as well as the high surface area to volume ratio, may make the fluffy Fe@*α*-Fe_2_O_3_ core-shell nanowire a promising candidate for the applications of the magnetic drug delivery, electrochemical energy storage, gas sensors, photocatalysis, and so forth.

## Background

In recent decades, the synthesis and properties of nanostructures have been greatly motivated both by a large number of potential applications and by fundamental questions about the physics of nanoscale magnetism. Comparing with other nanostructures, nanowires, especially ferromagnetic metal nanowires, have attracted more attention owing to their fundamental importance for various fields such as environmental remediation [1,2], biomedicine [3], magnetic sensors [4], and magnetic storage devices [5-7], etc. Furthermore, due to the special morphology, it usually exhibits many novel and unique physical characters, including magnetoimpedance (MI) effect [8], nanoscale confinement [9], and nanomagnetism [10], etc.

As the most commonly used magnetic element, iron (Fe)-based nanostructures have stimulated great interest for researchers in the past few decades [11,12]. However, one of the crucial problems in obtaining Fe nanostructures is that they commonly burn up when they are put into contact with air due to the strong activity of Fe. To avoid such a situation, encapsulating Fe nanostructures through the passivation with a Fe-oxide layer is adopted to both protect and stabilize the Fe nanostructures and thus form the core-shell morphology [13-15]. As a result, strong exchange magnetic coupling between the iron core and the oxide shell alters the magnetic anisotropy, giving rise to the modifications of the coercivity (*H*_*C*_) and the appearance of the exchange-bias (EB) effect [16-18]. The EB was first observed by Meiklejohn and Bean in oxide-coated Co particles in 1956 [19]. It is characterized by the horizontal shift of the hysteresis loops after the hybrid magnetic systems cooled down through the critical temperature in an external field [20]. For example, for the typical ferromagnetic (FM)/antiferromagnetic (AFM) hybrid magnetic system, the EB appears when the sample is cooled down from above the AFM N éel temperature in an external field. Up to now, the EB effect of Fe-based nanostructures, for example, zero-dimensional core-shell NPs of Fe/ *γ*-Fe_2_O_3 _[21], FeO/Fe_3_O_4 _[18], and Fe/Fe_3_O_4 _[22] have been systematically investigated. However, the physical origin of EB is still poorly understood. For the one-dimensional nanowires, the magnetic properties are even more complicated. The large aspect ratio, the high surface area to volume ratio, the shape anisotropy, and the interface play important roles in the magnetization dynamics of the core-shell structured systems. Therefore, the synthesis of one-dimensional Fe-based nanostructures and varying the magnetic properties via chemical control over the components could be important for the understanding of EB at the nanoscale level.

In this paper, Fe@*α*-Fe_2_O_3_ core-shell nanowires with novel fluffy-like *α*-Fe_2_O_3_ covered on the surface were synthesized. The structure, morphologies, and magnetic properties of the resulted nanowires have been comprehensively studied. It is found that the coercivity and the EB of the nanowires have been improved evidently by forming the Fe@*α*-Fe_2_O_3_ core-shell structure.

## Methods

The Fe@*α*-Fe_2_O_3_ nanowires were synthesized by a reaction between ferrous sulfate and sodium borohydride proposed by Tong et al. previously [23]. All reagents, such as ferrous sulfate heptahydrate (FeSO _4_·7H_2_O, AR) and sodium borohydride (NaBH_4_, AR), were obtained from commercial suppliers and were used without any further purification. A solution of 30.0 mL of 0.70 M NaBH_4_ was added into 60.0 mL of 0.050 M FeSO_4_ solution in a reaction flask while the solution was vigorously stirred. The reaction mixture was maintained at 60°C for up to 30 min with continuous stirring. The resulting black precipitates were separated from the solution by centrifugation at 4,000 rpm for 5 min, washed several times with deionized water and ethanol, and then dried in vacuum at 40°C for 24 h to obtain the as-synthesized product of the Fe@*α*-Fe_2_O_3_ nanowire. Annealing is the final heat treatment procedure. The annealing procedure was performed in a tube furnace under air atmosphere with a 6°C/min heating rate, and the sample was allowed to annealing at 380°C for 2, 4, 6, and 8 h, respectively. After the annealing process, the sample was cooled down to room-temperature. The cooling rate is also 6°C/min.

Structural analysis was performed by X-ray powder diffraction (XRD, D/max-2500) using the Cu Ka radiation (*λ* = 1.5406 Å). The microstructures, morphologies, and the elemental distribution of the nanowires were characterized by transmission electron microscopy (TEM, JEOL 2200F, Akishima-shi, Japan) operating at 200 kV. The magnetic properties were measured by a superconducting quantum interference device magnetometer (MPMS-5S) in magnetic fields up to 50 kOe and over the temperature range of 5 to 300 K.

## Results and discussion

**Figure 1:**
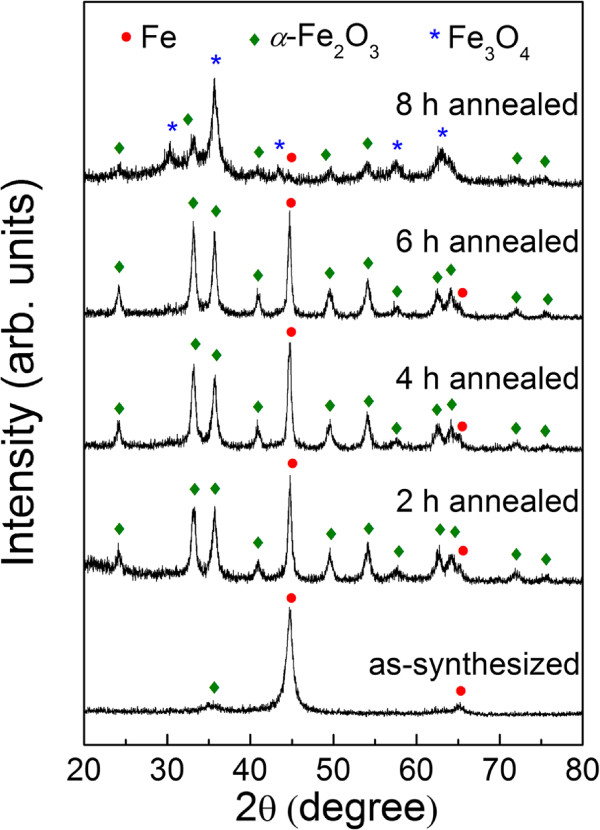
X-ray diffraction patterns of the as-synthesized and annealed samples.

Figure 1 displays the XRD patterns of the samples with different annealing time *T*_*A*_. It is found that all patterns are composed of two or three phases. For the as-synthesized sample, the diffraction peaks could be mainly indexed into the face-centered cubic (fcc) phase of irons. The lattice constant calculated from this XRD pattern is 2.862 Å, which is very close to the reported data (*a* = 2.860 Å, JCPDS file no. 87-0721). Besides, there is the hexagonal phase of hematite (*α*-Fe_2_O_3_, JCPDS card no. 33-0664, *a* = 5.036 Å and *c* = 13.749 Å). The relative intensity of XRD pattern of *α*-Fe_2_O_3_ phase is very low, indicating the very small amount of *α*-Fe_2_O_3_. No additional peaks corresponding to magnetite (Fe_3_O_4_) or maghemite (*γ*-Fe_2_O_3_) phase are observed in the as-synthesized sample. For the annealed sample, the relative intensity of the *α*-Fe_2_O_3_ peak increases evidently with increasing *T*_*A*_. However, for the 8-h annealed sample, it appears some magnetite (Fe_3_O_4_) impurity phase (JCPDS card no. 85-1436), which may be due to the lack of oxygen in the tube furnace for prolonged annealing process [24]. The average grain diameters can be estimated by the Scherrer formula. They are 9.1, 15.7, 18.0, and 20.9 nm for the as-synthesized, 2-h annealed, 4-h annealed, and 6-h annealed samples, respectively. It indicates that the grain size grows up with increasing *T*_*A*_. However, for 8-h annealed sample, the concentration of Fe is too low so that the grain size can hardly be estimated.

**Figure 2:**
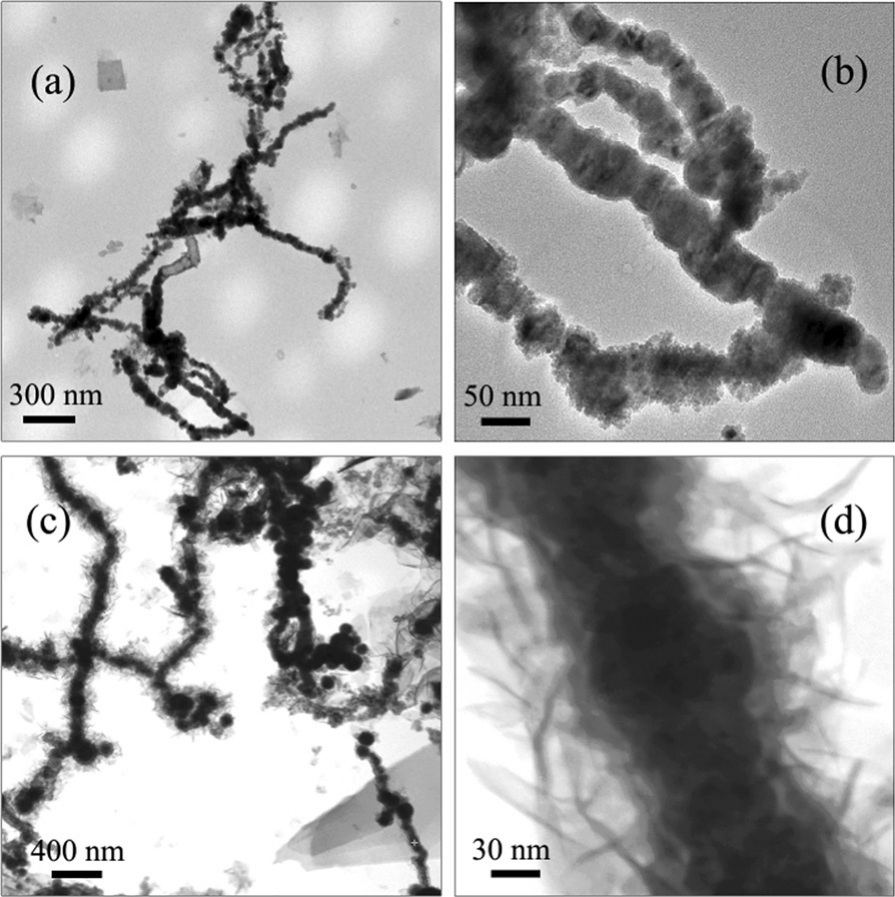
**TEM bright field images of Fe@Fe**_**2**_***O***_**3**_** core-shell nanowires.** Panels **(a)** and **(b)** indicate the as-synthesized sample. Panels **(c)** and **(d)** indicate the 4-h annealed sample.

Figure 2 shows the TEM bright field images of the samples before and after annealing. In Figure 2a,b, it shows that the as-synthesized sample is one-dimensional sphere-chain-like nanowire. The average diameter of the nanowire is approximately 70 nm, while the length is over 1 *μ*m. Besides, the TEM image in Figure 2b reveals the contrast between the gray edge and the dark center, suggesting the core-shell structure of the nanowires. The diameter of the core is more than 50 nm, while the thickness of the shell is less than 10 nm. Considering the facts that the metallic Fe is unstable in air and according to the XRD patterns shown in Figure 1, it can be inferred that the shell should be a thin layer of *α*-Fe_2_O_3_. Figure 2c,d shows the images of the nanowires after 4-h annealing. The annealed nanowires are also in core-shell structure with the diameter of core between 50 and 100 nm, which is not very uniform. Compared with the as-synthesized nanowires, the thickness of the shell is substantially increased after annealing. Moreover, it is interesting to find that after the 4-h annealing process, some novel fluffy-like phases germinate and grow on the surface of the oxidation layer as shown in Figure 2d. The morphology of the fluffy-like phases obtained here is similar to the urchin-like *α*-Fe_2_O_3_ reported in the literature [24], which were prepared via the oxidation of Fe spheres in air at the temperatures between 250°C and 400°C. It should be noticed that since the nanowires are oxidized in air and they are only composed of Fe and *α*-Fe_2_O_3_ phases as XRD patterns shown, we can infer that the fluffy-like phase here is the *α*-Fe_2_O_3_.

**Figure 3:**
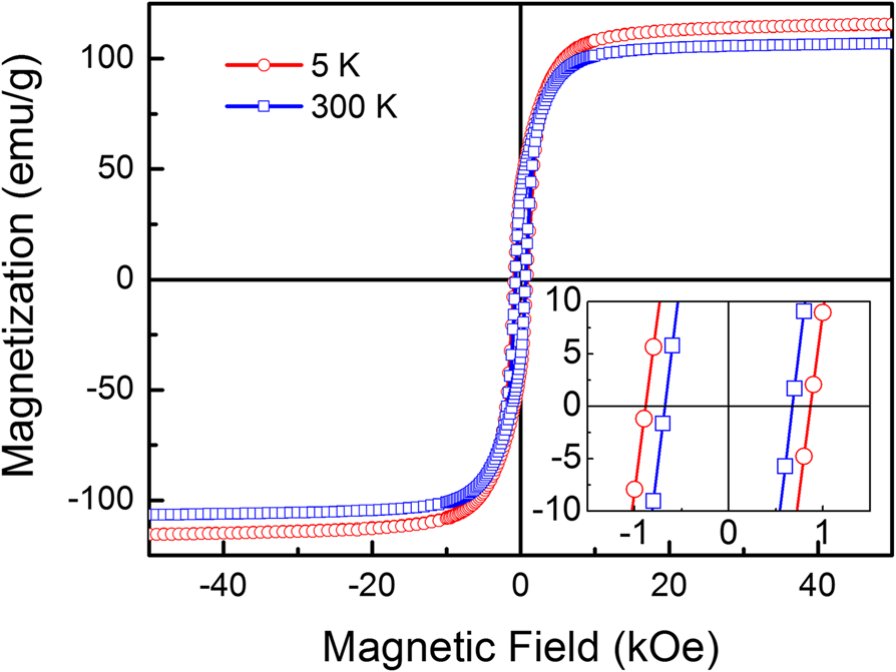
Hysteresis loops of the as-synthesized samples.

Figure 3 shows the hysteresis loops (MH) of the as-synthesized samples measured at 5 and 300 K. The 5 K saturation magnetization (*M*_*s*_) is approximately 116 emu/g, which is lower than that of the bulk Fe (218 emu/g) [25]. The decrease of *M*_*s*_ may be due to the existence of the AFM *α*-Fe_2_O_3_ at the surface of the nanowire as shown in the TEM image in Figure 2. It may also be caused by the defects and disorders in the nanostructure [26]. Interestingly, the *M*_*s*_ is still kept to be approximately 107 emu/g at 300 K, which means that the ferromagnetism can be extended to 300 K, indicating the improvement of the room temperature ferromagnetism stability over the thermal fluctuations. On the other hand, Figure 3 also shows that the *H*_*C*_ of the as-synthesized nanowire is approximately 878 Oe at 5 K. It decreases slightly to be approximately 684 Oe at 300 K. The values are remarkably higher than that of the bulk Fe (*H*_*C*_ approximately 0.9 Oe) [27]. It is known that in one-dimensional structure, the magneto-crystallize anisotropy is often lower than that of the shape anisotropy, so that the coercivity is mainly dominated by the shape anisotropy [28]. Thus, the large values of *H*_*C*_ in the as-synthesized nanowires may be attributed to the distinctive one-dimensional anisotropic structure of the magnetic nanowires with high shape anisotropy [29].

**Figure 4:**
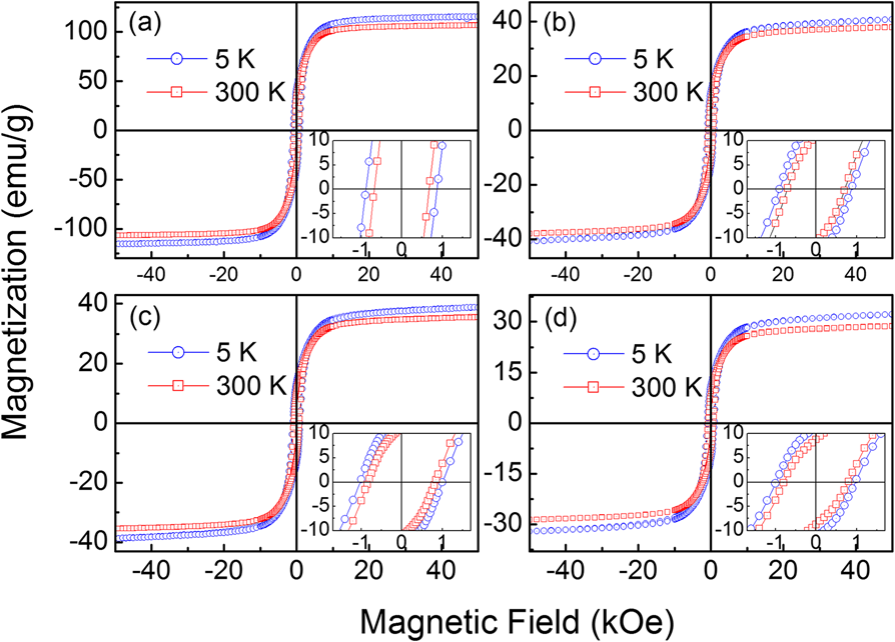
**The 5 and 300 K hysteresis loops measured after 10 kOe magnetic field cooled.** Panels **(a)**, **(b)**, **(c)**, and **(d)** are the as-synthesized, the 2-h annealed, the 4-h annealed, and the 6-h annealed nanowires, respectively. Inset displays detailed MH curves in low magnetic fields.

Figure 4 shows the MH curves of the novel fluffy Fe@*α*-Fe_2_O_3_ core-shell nanowires obtained by annealing the as-synthesized sample in air. The MH curve of the as-sythesized sample is also shown for comparison. The hysteresis loops at 5 K were obtained after cooling the sample from 300 to 5 K under a magnetic field of 10 kOe. It can be seen that the saturated magnetization is decreased with increasing *T*_*A*_, which indicates that the AFM *α*-Fe_2_O_3_ phase is increased after annealing and is in accordance with the XRD and TEM results. All samples in Figure 4 exhibit evident coercivity, which is defined by

(1) $$H_{C}=-(H_{\text{right}}-H_{\text{left}})/2.$$

Here, *H*_right_ and *H*_left_ are the positive and negative magnetic field values, respectively, where the magnetization goes through zero in the hysteresis loops. According to the 5 K hysteresis loop in the inset of Figure 4, the coercivity of the as-synthesized sample is approximately 881 Oe. After annealing the sample in air, the *H*_*C*_ increases distinctly. The 4-h annealed sample shows the maximum coercivity (approximately 1,042 Oe), which is much larger than that of the as-synthesized sample. Furthermore, the system exhibits EB with a horizontal shift along the negative magnetic field direction. The horizontal shift is a measurement of the exchange field (*H*_*E*_) given by

(2) $$H_{E}=-(H_{\text{right}}+H_{\text{left}})/2.$$

The *H*_*E*_ of the as-synthesized sample is only approximately 30 Oe measured at 5 K after a 10 kOe magnetic field cooling process. Similar to that of *H*_*C*_, *H*_*E*_ is also improved by annealing. The 4-h annealed sample shows the largest *H*_*E*_ of approximately 78 Oe at 5 K.

**Figure 5:**
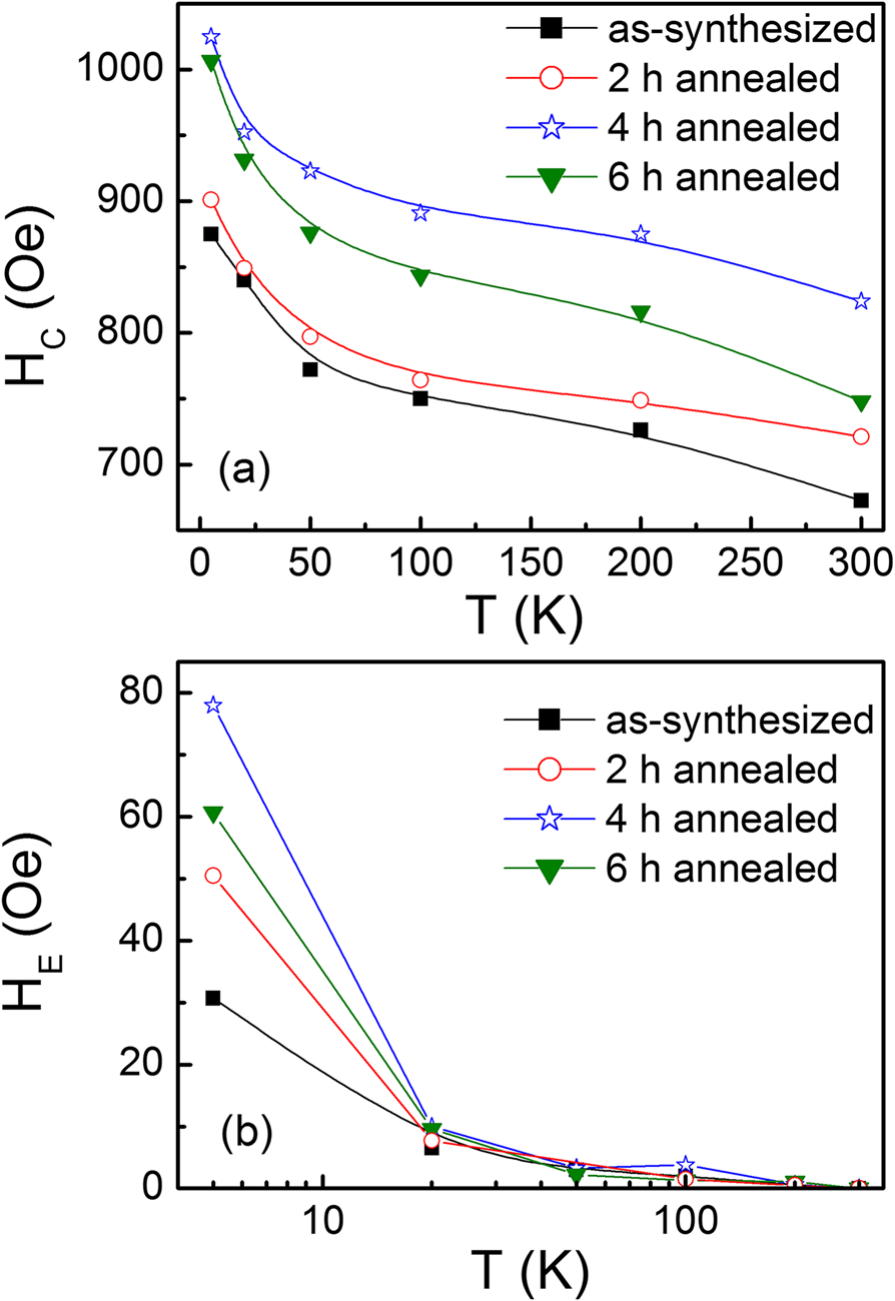
***H***_***C***_** and*H***_***E***_** values deduced from hysteresis loops at different temperatures.** Panels **(a)** and **(b)** are the temperature dependence of *H*_*C*_ and *H*_*E*_ for all samples. The straight lines are guides for the eyes.

The *H*_*C*_ values deduced from hysteresis loops at different temperatures (*T*) were plotted against *T* as shown in Figure 5a. It shows that *H*_*C*_ increases as the temperature decreases. At lower temperature of *T*<50 K, it increases rapidly. In the whole temperature range, the *H*_*C*_ of the annealed nanowires is higher than that of the as-synthesized sample. As shown in XRD and TEM images, the antiferromagnetic *α*-Fe_2_O_3_ phases formed at the surface of the nanowires. The appearance of the *α*-Fe_2_O_3_ phases will induce the additional unidirectional anisotropy energy due to the existence of exchange interactions between Fe core and *α*-Fe_2_O_3_ shell at the interface, and thus, the coercivity increases significantly than that of the pure Fe due to the spin drag effect for the unpinned uncompensated spin at the interface [30]. At a certain measuring temperature, the *H*_*C*_ increases with increasing *T*_*A*_, reaching the maximum at *T*_*A*_ = 4 h. The increase of *H*_*C*_ with *T*_*A*_ may be caused by several reasons. First, the as-synthesized nanowires have high intrinsic stress due to the rapid chemical reactions. The anisotropy induced by stress may compete directly with shape anisotropy, which will decrease the coercivity. The annealing process will reduce the internal stress, so the coercivity is improved [31]. Second, the AFM thickness at the outside of the nanowires is increased evidently by annealing, which will increase the AFM anisotropy energy, and thus enhance the drag effect for the interfacial unpinned uncompensated spins [18]. It is noticeable that the *H*_*C*_ decreases with further increasing *T*_*A*_ above 4 h. This may be because that when the AFM thickness further increases, the AFM anisotropy energy is increased and the pinning effect is further enhanced. At this time, the amounts of the interfacial unpinned uncompensated spins, which contribute to the coercivity, may decrease and reduce the *H*_*C*_.

Figure 5b displays the temperature dependence of *H*_*E*_ for different nanowires measured under the cooling magnetic field of 10 kOe. It can be seen that for all samples, *H*_*E*_ decreases monotonically with increasing temperature and becomes negligibly small above the temperature of 50 K. At a certain temperature, *H*_*E*_ increases first with increasing *T*_*A*_ and then decreases with further increasing *T*_*A*_, exhibiting a maximum at *T*_*A*_ = 4 h. The enhancement of *H*_*E*_ with increasing *T*_*A*_ may be mainly because of the increase of the thickness of AFM Fe_2_O_3_ shell at the surface of the nanowires [18,32]. While the decrease of the *H*_*E*_ for 6-h annealed sample is rather complicated. This may depend on the microstructure, for example, the change of the AFM domain structure [18]. This phenomenon has also been found in other exchange bias systems [32-34].

**Figure 6:**
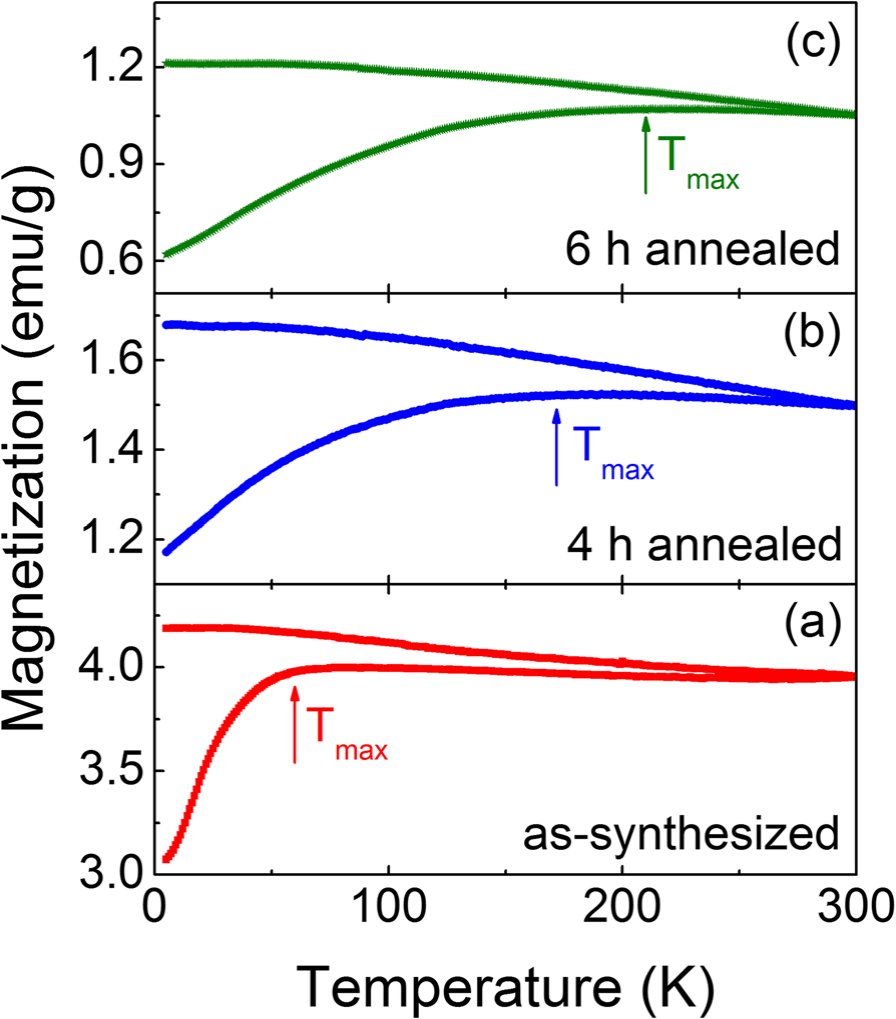
ZFC/FC magnetization curves measured under an applied magnetic field of 200 Oe.

In order to gain the further insight into the magnetic properties of Fe@*α*-Fe_2_O_3_ nanowires, zero field-cooled (ZFC) and field-cooled (FC) magnetization curves were investigated. During the ZFC process, the sample was first cooled down from room temperature (RT) to 5 K under a zero magnetic field. Then, a magnetic field of 200 Oe was applied, and the magnetic moment was recorded as the temperature increases from 5 to 300 K to obtain the ZFC curve. For simplicity, the magnetic moment was then directly measured from 300 to 5 K to get the FC curve. Figure 6 shows the ZFC/FC curves of three typical samples, i.e., the as-synthesized sample, the sample annealed for 4 h, and the sample annealed for 6 h. For the as-synthesized sample in Figure 6, the irreversibility exists in the whole temperature range. The ZFC magnetization increases rapidly from 5 to 65 K and then decreases slightly with increasing *T*, exhibiting a broad peak (*T*_max_ approximately 65 K). The FC magnetization decreases continuously as temperature increases from 5 to 300 K. These behaviors of ZFC/FC curves are related to a superparamagnetic behavior of the crystal grains whose blocking temperatures are widely distributed. The distribution of the blocking temperature indicates that the energy barriers, which are contributed by the anisotropy energy and the dipolar interactions, have wide distributions. This distribution may be caused by the distribution of the crystal grain sizes as TEM images show in Figure 2. Similar to the as-synthesized sample, the 4-h annealed sample also exhibits the superparamagnetic behavior. The bifurcations are also higher than 300 K. The most important feature is that the ZFC magnetization shows a maximum around 170 K, which is higher than 65 K of the as-synthesized sample. The fact that the block peak shifted to the higher temperature implies that the strength of the energy barriers is increased to overcome the thermal fluctuations. For the 6-h annealed sample, the peak temperature is further improved, indicating that the strength of the energy barriers is further increased.

## Conclusions

In conclusion, the Fe@*α*-Fe_2_O_3_ nanowires have been synthesized using the chemical method. Some novel fluffy-like *α*-Fe_2_O_3_ grows on the surface of the nanowires through the post-annealing in air. The coercivity of the as-synthesized nanowires is above 684 Oe in the temperature range of 5 to 300 K, which is significantly higher than that of the bulk Fe. Through the annealing process in air, the coercivity and the exchange field are evidently improved. Both the coercivity and the exchange field increase with increasing *T*_*A*_ and reach their maximum values of 1,042 and 78 Oe, respectively, at *T*_*A*_ = 4 h. The magnetic measurements show that the effective anisotropy is increased with increasing the thickness of the *α*-Fe_2_O_3_ by annealing. The large values of coercivity and exchange field, as well as the high surface area to volume ratio, may make the fluffy Fe@*α*-Fe_2_O_3_ core-shell nanowire a promising candidate for the applications of the magnetic drug delivery, electrochemical energy storage, gas sensors, photocatalysis, and so forth.

## Competing interests

The authors declare that they have no competing interests.

## Authors’ contributions

XC carried out the synthesis of the nanowire and participated in the data analysis. WW and XZ measured the magnetic properties. LL carried out the X-ray diffraction. YC and HL participated in the design and coordination of the study, analyzed the experimental data, and wrote the manuscript. SD carried out the TEM measurements. RZ participated in the data analysis and modified the manuscript. All authors read and approved the final manuscript.
